# Does teaching social and communicative competences influence dental students’ attitudes towards learning communication skills? A comparison between two dental schools in Germany

**DOI:** 10.3205/zma001165

**Published:** 2018-05-15

**Authors:** Nora V. Lichtenstein, Rainer Haak, Isabelle Ensmann, Houda Hallal, Jana Huttenlau, Katharina Krämer, Felix Krause, Jan Matthes, Christoph Stosch

**Affiliations:** 1 University of Cologne, Faculty of Medicine, Office of the Vice Dean for Teaching and Studies, Cologne, Germany; 2 University of Leipzig, Department of Cariology, Endodontology and Periodontology, Leipzig, Germany; 3 University of Cologne, Department of Operative Dentistry and Periodontology, Cologne, Germany; 4 University of Cologne, Department of Psychiatry, Cologne, Germany; 5 University of Cologne, Department of Pharmacology, Cologne, Germany

**Keywords:** Communication Skills Attitude Scale (CSAS-D), social skills, communication skills, longitudinal curriculum, dental education

## Abstract

**Introduction:** Teaching social and communicative competences has become an important part of undergraduate dental education. The aim of this study was to explore the influence of a longitudinal curriculum, addressing social and communication skills, on dental students’ attitudes towards learning these skills.

**Material and methods: **Data on the attitudes towards learning communication skills were collected at two German universities and compared in a cross-sectional survey. 397 dental students were included, 175 students attended a longitudinal curriculum addressing social and communicative competences while 222 students did not. The dental students’ attitude towards learning communication skills was measured by a German version of the Communication Skills Attitude Scale (CSAS-D).

**Results: **Dental students who participated in a longitudinal communication curriculum had significantly lower negative attitudes towards learning communication skills than students who did not attend such courses. Differences in positive attitudes could not be found. Significant interaction effects were found for the factors gender and section of study: female students in the clinical section of their study who participated in the longitudinal curriculum reported higher positive attitudes and lower negative attitudes compared to female students in the preclinical section of study.

**Conclusion: **The results of this study indicate that a longitudinal curriculum addressing communication skills can enhance positive and reduce negative attitudes towards learning communication skills. More longitudinal data is needed to explore to what extent gender affects development of communication skills and how students’ attitudes towards learning communication change in the long run.

## Zusammenfassung

**Einleitung: **Die Vermittlung von sozialen und kommunikativen Kompetenzen ist zu einem wichtigen Bestandteil der zahnmedizinischen Ausbildung geworden. Das Ziel dieser Studie war es, den Einfluss eines longitudinalen Curriculums für soziale und kommunikative Kompetenzen auf die Einstellung von Zahnmedizinstudierenden gegenüber dem Erlernen dieser Kompetenzen zu untersuchen.

**Material und Methode: **Daten über die Einstellungen gegenüber dem Erlernen von kommunikativen Fähigkeiten wurden an zwei deutschen Universitäten in einer Querschnittsbefragung erhoben. 397 Studierende der Zahnmedizin wurden in die Untersuchung einbezogen, 175 Studierende nahmen an einem longitudinalen Curriculum für soziale und kommunikative Kompetenzen teil während 222 Studierende nicht daran teilnahmen. Die Einstellungen der Zahnmedizinstudierenden gegenüber dem Erlernen von kommunikativen Fähigkeiten wurde mit einer deutschen Version der Communication Skills Attitude Scale (CSAS-D) erhoben.

**Ergebnisse: **Zahnmedizinstudierende, die an einem longitudinalen Kommunikationscurriculum teilnahmen, hatten signifikant niedrigere negative Einstellungen gegenüber dem Erlernen kommunikativer Fähigkeiten als Studierende, die nicht an solchen Kursen teilgenommen haben. Unterschiede in den positiven Einstellungen konnten nicht gefunden werden. Signifikante Interaktionseffekte gab es bei den Faktoren Geschlecht und Studienabschnitt: Weibliche Studierende im klinischen Studienabschnitt, die an dem longitudinalen Curriculum teilgenommen hatten, berichteten höhere positive und niedrigere negative Einstellungen verglichen mit weiblichen Studierenden im vorklinischen Studienabschnitt.

**Schlussfolgerungen: **Die Ergebnisse dieser Untersuchung deuten darauf hin, dass ein longitudinales Kommunikationscurriculum dazu beitragen kann, die positiven Einstellungen gegenüber dem Erlernen von kommunikativen Fähigkeiten zu verstärken und die negativen Einstellungen zu reduzieren. Weitere longitudinale Daten sind nötig, um zu untersuchen, in welchem Umfang das Geschlecht einen Einfluss auf die Entwicklung von kommunikativen Fähigkeiten hat und wie sich die Einstellungen der Studierenden gegenüber dem Erlernen von Kommunikation auf lange Sicht verändern.

## Introduction

The success of dental treatment is distinctively influenced by the dentist-patient-relationship [1]. This relationship in turn depends in large part on the quality of the communication between dentist and patient [2]. Since communication skills of dental students do not improve automatically with contact to patients in clinical treatment courses [3], teaching communication skills to dental students is increasingly recognized as an important part of dental education. Early exposure to the concept of dentist-patient-interaction is particularly important in dentistry because dental students are involved in treating patients within the first years of the clinical training. Even though the importance of social and communication skills for the dental profession has been widely accepted, there is no uniform curriculum for social and communicative competences in dental schools in Germany so far. Surveys show that most dental schools in Germany include social and communication and social skills in their curriculum, but only a few established longitudinal curricula [4], [5]. Learning objectives for training of communication and social skills during the academic studies in dental school have been outlined by the Association for Dental Education in Europe (ADEE) [6], in the Basler Consensus-Statement [7] and the Health Professions Core Communication Curriculum (HPCCC) [8]. 

Based on these recommendations an evidence-based longitudinal curriculum addressing the dental-patient communication has been developed and implemented at the University of Cologne in 2009. This longitudinal curriculum includes multiple interrelated course elements so that students deal with different aspects of the dentist-patient communication multiple times during their academic studies (for further information see [9]). The program was funded by a governmental quality-enhancement-pact. 

In view of the importance of teaching both, technical expertise and attitudinal approaches to communication [10], [11], the courses include specific skills like active listening or structuring a patient-dentist-conversation as well as developing professional attitudes. Attitudes in general play an important role in explaining and predicting human behavior [12], [13], [14]. They develop slowly, but remain quite stable over time [13], [15]. Therefore, developing positive attitudes towards communication skills during the time at university is as important as acquiring knowledge of communication theory and the actual training of specific communication skills to make sure that students are well prepared for interacting with patients and dental teams. However, according to several studies students’ attitudes towards learning communication skills become more negative during their academic studies [16], [17], [18]. These findings suggest that additional efforts are required to maintain students’ positive attitudes to learning communication skills [16]. The mere attendance of communication courses does not necessarily lead to the development of positive attitudes towards communication learning: While some studies report that communication trainings enhance students’ positive and reduce their negative attitudes towards learning communication [19], [20], there are others showing that positive attitudes decline after attending a communication course [21], [22]. Most of the studies, however, measured the effects on attitude development after attending only a single communication course. So far there are only few studies focusing on dental students [5], [23] and even less studies addressing the influence of more than one communication course on dental students’ attitudes towards learning communication skills [24]. 

Within the study framework of dental medicine at the University of Cologne, a longitudinal communication curriculum was implemented, where students have to attend various communication courses throughout their whole academic studies. The aim of the present study was to determine to what extent such a longitudinal communication curriculum influences students’ attitudes towards learning communication skills: We expected dental students, who attended a longitudinal communication curriculum, to report more positive and less negative attitudes towards learning communication skills compared to non-attendees (see table 1 (Tab. 1)).

## Materials and methods

To explore whether the longitudinal curriculum on communication skills influences students’ attitudes towards learning communications skills, the appraisal of student populations from two dental schools in Germany were compared. Students at the dental school in Cologne participated in communication courses as part of a longitudinal communication curriculum. These courses are based on the competences for dentists defined by the Association for Dental Education in Europe (ADEE) [6] and include different aspects of communication and social skills. The preclinical communication courses focus on the basics of communication like establishing an initial rapport, active listening and giving and receiving constructive feedback. In the clinical section of study the communication courses focus on shared decision making and different aspects of teamwork and diversity-sensitive communication (for further information see [9]). Students at the University in Leipzig did not attend such courses, due to the fact that no such communication curriculum was implemented at the time of evaluation. 

To measure the attitudes towards learning communication a German version of the communication skills attitude scale for students (CSAS) developed by Rees et al. [25] was used (CSAS-D, Speidel et al [26]). This questionnaire measures students' attitudes towards learning communication skills in medical school. There is no German questionnaire that refers to dental students solely. Laurence et al. [27] developed an English version of the CSAS for dental students, where the terms “medical“ and “physician“ were exchanged by “dental“ and “dentist“. A similar adaption in the German version is unnecessary since the German term “medical” applies to both, physicians and dentists. The CSAS-D consists of 26 items with two subscales, each including 13 items. The items are statements addressing learning of communication skills during the time of medical studies at University. The first subscale addresses positive attitudes to learning communication skills (positive attitude scale=PAS) and the second subscale addresses negative attitudes to learning communication skills (negative attitude scale=NAS). The items of the PAS are clustered together in three groups:

1. students' beliefs that communication skills learning would facilitate their interpersonal skills with both their colleagues and with patients;
2. students' beliefs that communication skills learning was fun and interesting; and
3. students' beliefs that communication skills learning was important within a medical context […] ([25], p.145).

The items of the NAS clustered together in four groups:

1. “[…] (medical students' negative attitudes towards communication skills learning as a social science subject; […];
2. students' apathy towards learning communication skills, […];
3. students' negative beliefs that communication skills learning was difficult to take seriously; and
4. students' negative attitudes towards communication skills assessment.” ([25], p.145).

The items are rated on a 5-point Likert scale (1=“strongly disagree” to 5=“strongly agree”, see attachment 1 ). Means of the two subscales were calculated and compared. Internal consistency of the scales was found to be acceptable to good, with Cronbach's alpha of .72 for NAS and .83 for PAS. 

The Ethics Committee of the University of Cologne raised no concerns regarding this study or the publication of the results (reference No. 16-139). The survey was conducted during the 2012 summer semester. Students were asked to anonymously fill out the questionnaire and to provide additional information about their age, gender and semester. To avoid additional time expenditure this was done during regular courses. Participation in the study was voluntarily and the students were assured that their responses were confidential and would have no bearing on their overall academic assessment. All data were collected and analyzed anonymously. The questionnaires from Leipzig were sent to Cologne for data analysis. 

In total 140 (female=106) questionnaires in Cologne (35% of the local dental student population at the time of the survey) and 212 (female=133) in Leipzig (56% of the local dental student population at the time of the survey) were filled out completely and included in the study. Since the students were asked to fill out the questionnaire during regular course time, the survey was conducted only at courses where members of the study had access to. Due to technical and structural reasons it was not possible to ask the entire dental student population. The participants in Cologne primarily came from the second year (29,3%), in Leipzig from the third year (29,2%) (see table 2 (Tab. 2)). Due to these differences and the structure of the dental training in Germany, it was decided to summarize all data from the respective preclinical semesters and compare them to the data from the respective clinical semesters. There was no significant difference in age (t (1, 344)=.380, p>.05) between participants from Cologne (M=23.98; SD=3.607) and Leipzig (M=23.84; SD=2.886). 

A three-factorial ANOVA with the factors *university, section of study* and *gender* was used to analyze the data. Planned simple comparisons (Bonferroni-corrected) were computed to break down interaction effects. Cohen’s correlation coefficient (r) is used to interpret the effect sizes (small effect 0.1≤r<0.3, medium 0.3≤r<0.5, large 0.5≤r) [28]. All data were analyzed using IBM SPSS Statistics, version 22.

## Results

Overall, there was a significant main effect of the factor *section of study* for PAS [F(1,344)=14.719, p=.000, r=.203], but not for NAS [F(1,344)=1.575, p=.210]. Students from the clinical courses (M=3.7; SD=.5) had lower PAS scores than students from the preclinical courses (M=3.8; SD=.5).

On the other hand, we found a significant main effect of the factor *University* for NAS [F(1,344)=13.226, p=.000, r=.192], but not for PAS [F(1,344)=1.317, p=.252]. Dental students, who participated in the longitudinal communication curriculum in Cologne, had significantly lower NAS scores (M=2.1; SD=.4) than the dental students in Leipzig, who did not attend such courses (M=2.3; SD=.4).

Also there was a significant main effect of the factor *gender* regarding NAS [F(1,344)=14,729, p=.000, r=.203], but not PAS [F(1,344)=.611, p=.435]. Female students (M=2.2; SD=.4) had lower NAS scores than male students (M=2.4; SD=.5).

Additionally significant three-way interactions of the factors *university, section of study* and *gender* were found for both subscales PAS [F(1,344)=10.1, *p*=.002, *r*=.169] and NAS [F(1,344)=5.249, *p*=.023, *r*=.123]. Simple comparisons (see table 3 (Tab. 3)) show that female students in Cologne were the only participants whose PAS scores are significantly higher in the clinical courses (M=3.9; SD=.4) than in the preclinical courses (M=3.7; SD=.4). All other subgroups show converse results: Male students in Cologne as well as female and male students in Leipzig reported lower PAS scores in the clinical courses than in the preclinical section of study. There is a similar trend looking at the NAS scores (see table 4 (Tab. 4)): Female students in Cologne reported lower NAS scores in the clinical courses (M=2; SD=.3) than in the preclinical courses (M=2.3; SD=.5). There are no significant differences between preclinical and clinical section of study in the other subgroups.

## Discussion

So far, studies addressing dentals students’ attitudes towards learning communication skills mainly focused on the effects of attending a single communication course [5], [23]. In the current study, we were able to compare students, who participated in multiple communication courses as a part of a longitudinal curriculum teaching communication skills, to students who did not attend such long-term courses. The goal of this study was to explore whether students participating in various interrelated communication courses teaching communication skills have higher positive and lower negative attitudes towards learning communication skills compared to students who did not attend such courses. The results show that dental students who participated in a longitudinal curriculum addressing communication skills reported significantly lower negative attitudes than students who did not attend such courses. Corresponding expected higher positive attitudes, however, could not be found.

Our results mainly indicate an effect of the factor *section of study* on the development of attitudes towards learning communication skills. This is in accordance to previous findings of a loss of medical students’ positive attitudes and an increase of negative attitudes towards communication skills during their academic studies [16], [17], [18]. Similar results were recently reported in a study with dental students [23]. Several explanations for this phenomenon are provided. Some authors suggested a general loss of empathy during dental as well as medical academic studies [29], [30] which could also imply a decline of positive attitudes toward communication skills. Sherman & Cramer [29] argued that this loss of empathy could be linked to a focus shift during the clinical section of studies. Increased technical demands during the intensive clinical training as preparation for the final exams could lead to a neglect of presumably less essential skills and behaviors like communication skills. Furthermore, the general loss of empathy can be seen as a part of a specific socialization process in medical education. In this context staying detached from patients’ suffering is understood as a protection from distress [31], [32]. Others argue that the described loss of empathy is an exaggeration due to uncritical interpretation of data gathered with self-report instruments. The low response rates, small effect-sizes and the limited validation of self-report instruments are not sufficiently taken into account when interpreting the results according to Colliver et al. [33]. Additionally, Rees et al. [18] found that students who thought they need to improve their communication skills tend to have higher PAS and lower NAS scores. A decline in positive attitudes towards learning communication skills during proceeding academic studies could thus indicate that students do not see the need for additional communication learning and do not value the opportunity offered by these courses because they already regard themselves as good enough at communicating with patients and coworkers. In order to further investigate this hypothesis, students’ perceived and objectively measured communication skills (e.g. OSCE marks) should be assessed in future studies. It is also conceivable that high positive attitudes at the beginning of medical school are a result of a social desirability bias: Students in their first semester are more likely to give socially desirable answers than students who are more experienced [16]. Finally, it is possible that the decline in positive attitudes towards learning communication skills is a result of a negative perception of the way these skills are taught [16] rather than a lack of appreciation for their importance. Even though students’ evaluation of the communication courses in Cologne have been positive so far [9] further evaluation is needed to exclude this explanation. 

Interestingly, our results indicate that the factor* gender* interacts with the factor *section of study* on attitude changes towards learning communication skills in a longitudinal curriculum: In Cologne, female students in the clinical section of their study who participated in the longitudinal curriculum reported higher positive attitudes and lower negative attitudes compared to female students in the preclinical section of study. This indicates that a longitudinal curriculum teaching communication skills, as implemented at the University of Cologne, could counteract the decline in positive attitudes towards communication skills during academic studies and thus influence students’ attitudes in a favorable way. It is notable that this result only refers to the female participants in this study. Male students who also attended the communication curriculum answered differently: they reported significantly lower PAS scores in the clinical section of study compared to the preclinical section of study and their NAS scores do not differ significantly. Gender effects associated with the CSAS reported in the literature are inconsistent. The majority of studies found gender effects comparable to those observed in this study, suggesting that female students have higher PAS and lower NAS scores than male students [17], [18], [19], [23], [27], [34]. Several studies, however, failed to find a significant difference between male and female participants [16], [35]. Cleland et al. [17] found that female students ranked their competences in communication skills lower than their male students. Taking into account the considerations of Rees et al. [18], this could lead to higher PAS and lower NAS scores, because female students are more likely to think their communication skills need improvement and therefore value the opportunity of attending communication courses. Furthermore, possible differences in the way female and male students learn are discussed as a factor influencing students’ attitudes towards learning communication skills [17]: It has been argued that the learning methods used in the communication courses like role plays or video feedback could be more suited to the learning style of females. Regarding the influence of role modeling, it has to be considered, that the teaching staff from the core-team in teaching communication skills is female and there might be a lack of male role-models as well. In addition, different speeds of learning could lead to different PAS and NAS scores. Apparently, overall, male students are slower at learning communication skills than female students [36]. Taken together, results of the present study and previous research on gender effects emphasize the importance of further investigating differences in attitudes towards learning communication skills between female and male students. This way, potential gender issues can be taken into account when planning and conducting communication courses as suggested by Rees and Sheard [21]. 

Finally, it should be considered that besides learning communication skills explicitly in the courses, students constantly interact with teachers, classmates and patients outside the classroom. Additionally, they observe interactions between faculty members or teachers and patients. These experiences are part of their daily routine at university and also shape their attitudes to communication learning. These influences, as parts of the “hidden curriculum”, should be explored in greater depth [16]. 

Methodological limitations must be taken into consideration when interpreting the results of the present study. Since the students cooperated voluntarily and the return rate in Cologne was lower than in Leipzig, a selection bias could have influenced the results, especially in Cologne. Interpretations of the differences between the preclinical and clinical section of study have to be interpreted carefully due to the cross sectional data and the heterogenic distribution of participants according to gender and section of study (more female and preclinical students in Cologne than in Leipzig). Longitudinal surveys are needed to describe the development of students’ attitudes during their academic studies more accurately and reduce possible cohort biases. Even though the reported results are statistically significant, the small effect sizes have to be taken into account when interpreting the results. All participants in the study reported rather high positive attitudes (between “agree” and “strongly agree”) and moderate negative attitudes (between “neutral” and “disagree”). With regards to absolute means and the small effect sizes, it remains unclear whether the significant differences truly imply a change in attitudes and whether this has an impact on students’ behavior. Based on these considerations, we agree with Martin et al. [37] to go beyond self-assessment questionnaires and use a multidimensional approach that also includes paper cases and observation to fully assess attitude changes in future research.

Our study was conducted in 2012. Since then the acceptance of including communication skills training in the dental education seems to have increased, as for example indicated by the respective learning goals in the National Competence Based Catalogue of Learning Objectives for Dental Education [38]. On the other hand, an amendment of the Dental Licensure Act that might emphasize communication in dental education is still in preparation. The University of Leipzig also implemented a communication curriculum by now. At the University of Cologne additional coursed were integrated in the already existing curriculum. Whether these changes have an impact on the students’ attitudes towards learning communication skills should be addressed in further studies. 

## Conclusions

There are many surveys exploring the effects of communication skills training in medical and dental education, but only few address the influence of communication skills training on students’ attitudes towards learning these skills. The current study contributes to close this gap and reveals implications for educational practice and future research. The results show that at least one subgroup of the participants in the study reported attitudes towards learning communication skills in a favorable way: female students in the clinical section, who participated in the longitudinal communication curriculum, reported higher positive and lower negative attitudes compared to female students in the preclinical section of study. This can be taken as a hint towards longitudinal communication curricula being effective and thus counteracting the often observed decline in positive attitudes towards communication skills during academic studies. 

An important aspect for future research is to explore to what extent gender affects learning of communication skills. Such information will be useful when planning communication courses. Also, the effects of longitudinal curricula on the development and change of attitudes towards communication skills should be examined in longitudinal studies. So far studies only investigated pre and post comparisons of students’ attitudes towards communication skills after attending a single communication course during the preclinical section of their study. It is necessary to gain more longitudinal data on how attending communication courses influence students’ attitudes towards communication learning in the long term in order to further improve and shape the communication curriculums at dental schools. 

## Competing interests

The authors declare that they have no competing interests. 

## Supplementary Material

CSAS-D Einstellung zum Erlernen von kommunikativen Fähigkeiten - only in german

## Figures and Tables

**Table 1:**
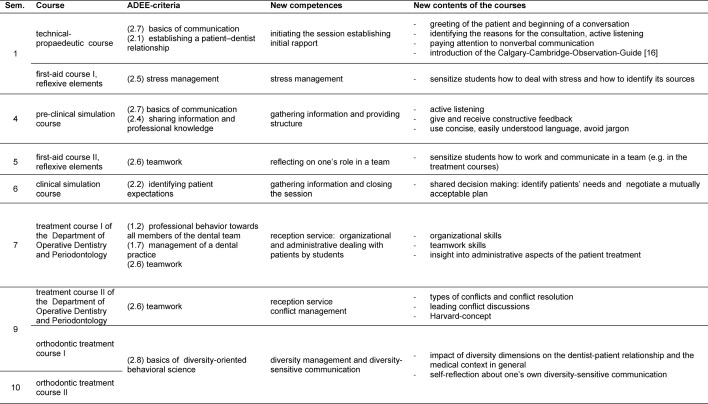
Overview of the course elements of the Longitudinal Curriculum of Social and Communicative Competences for Dentists (LSK-Dent) referring to the respective ADEE-criteria

**Table 2:**
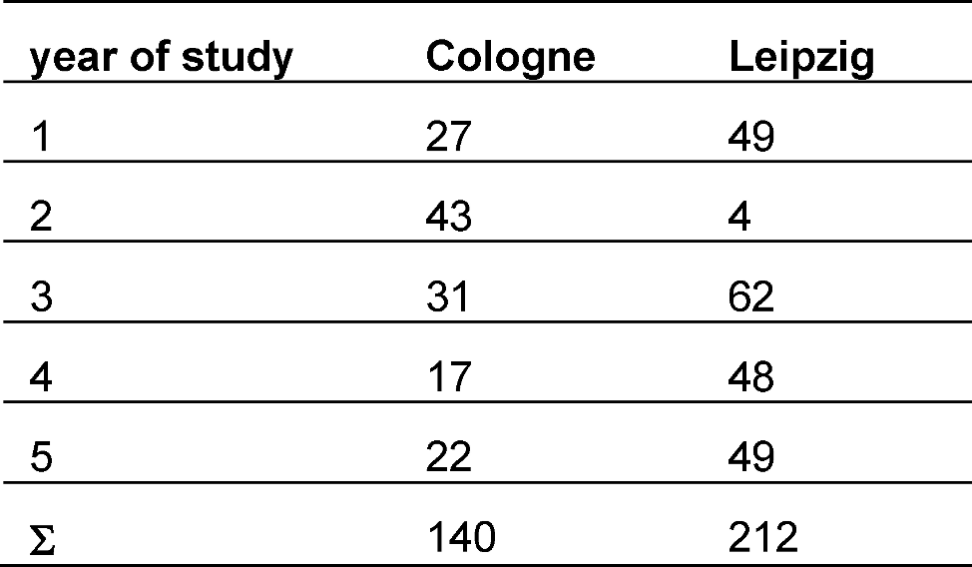
Distribution of participants in the study

**Table 3:**
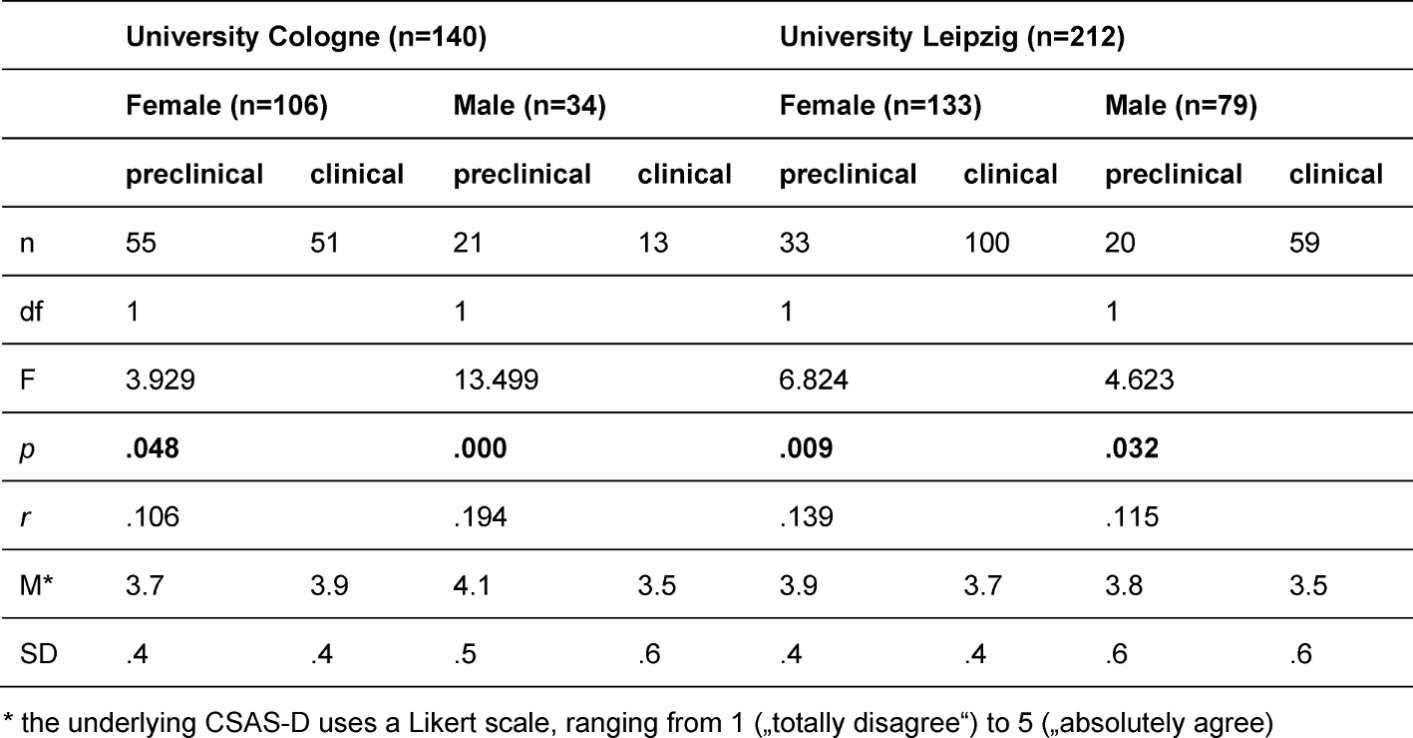
Simple comparisons, means and standard deviations of the interaction effect of University, section of study and gender for positive attitude scale (PAS)

**Table 4:**
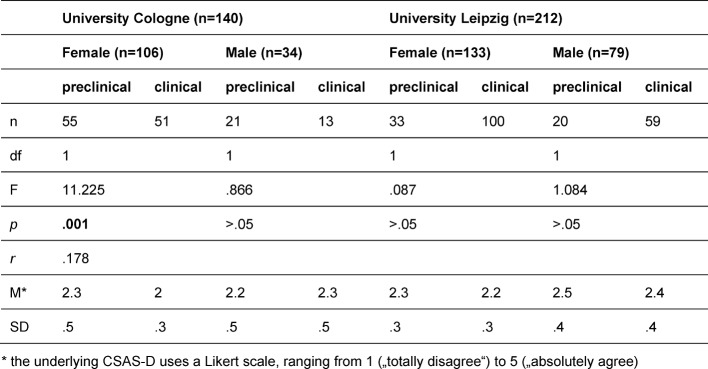
Simple comparisons, means and standard deviations of the interaction effect of University, section of study and gender for negative attitude scale (NAS)
