# Supplementary material for: Does teaching social and communicative competences influence dental students’ attitudes towards learning communication skills? A comparison between two dental schools in Germany
Source: GMS J Med Educ. 2018 May 15;35(2):Doc18. doi: 10.3205/zma001165 (PMC6022579; doi:10.3205/zma001165)
Supplement: CSAS-D Einstellung zum Erlernen von kommunikativen Fähigkeiten - only in german [file JME-35-18-s-001.pdf]

CSAS-D Einstellung zum Erlernen von kommunikativen Fähigkeiten[1]

Bitte lesen Sie die folgenden Aussagen zum Lernen kommunikativer Fähigkeiten. Geben Sie an ob Sie zustimmen oder widersprechen, indem Sie die am ehesten zutreffende Antwort markieren.

	**Aussage**	**stimme über-haupt nicht zu**	**stimme nicht zu**	**neutral**	**stimme zu**	**stimme sehr zu**
**1**	Um ein guter Arzt zu sein muss ich gute kommunikative Fähigkeiten haben.					
**2**	Ich sehe keinen Sinn darin, kommunikative Fähigkeiten zu erlernen.					
**3**	Niemand wird im Examen durchfallen weil er schlechte kommunikative Fähigkeiten besitzt.					
**4**	Meine kommunikativen Fähigkeiten zu entwickeln ist ebenso wichtig wie mein medizinisches Wissen zu entwickeln.					
**5**	Das Erlernen kommunikativer Fähigkeiten hilft mir, Patienten zu respektieren.					
**6**	Ich habe keine Zeit kommunikative Fähigkeiten zu erlernen.					
**7**	Das Erlernen kommunikativer Fähigkeiten ist interessant.					
**8**	Ich habe keine Lust, an Lehrveranstaltungen zu kommunikativen Fähigkeiten teilzunehmen.					
**9**	Das Erlernen kommunikativer Fähigkeiten hilft mir, meine Teamfähigkeit zu fördern.					
**10**	Das Erlernen kommunikativer Fähigkeiten hat meine Fähigkeiten mit Patienten zu kommunizieren verbessert.					
**11**	Der Unterricht kommunikativer Fähigkeiten spricht das Offensichtliche aus und verkompliziert es dann.					
**12**	Das Erlernen kommunikativer Fähigkeiten macht Spaß.					

[1] Deutsche Übersetzung der Communication Skills Attitude Scale von C. Rees et al.

© V. Speidel, L. Willms, H. Greß & V. Köllner, Homburg/Saar

	Aussage	stimme über-haupt nicht zu	stimme nicht zu	neutral	stimme zu	stimme sehr zu
**13**	Das Erlernen kommunikativer Fähigkeiten ist zu einfach.					
**14**	Das Erlernen kommunikativer Fähigkeiten hilft mir, meine Kollegen zu respektieren.					
**15**	Ich finde es schwierig, Informationen zu kommunikativen Fähigkeiten zu vertrauen, die ich von nicht-klinischen Dozenten erhalte.					
**16**	Das Erlernen kommunikativer Fähigkeiten hilft mir, Patientenrechte in Bezug auf Schweigepflicht und Einverständniserklärung (informed consent) anzuerkennen.					
**17**	Der Unterricht kommunikativer Fähigkeiten hätte ein besseres Image, wenn es mehr wie ein wissenschaftliches Thema klingen würde.					
**18**	Als ich mich für das Medizinstudium bewarb, hielt ich es für eine richtig gute Idee, kommunikative Fähigkeiten zu lernen.					
**19**	Ich brauche keine guten kommunikativen Fähigkeiten um Arzt/Ärztin zu sein.					
**20**	Es fällt mir schwer zuzugeben, dass ich etwas Probleme mit meinen kommunikativen Fähigkeiten habe.					
**21**	Ich finde es wirklich sinnvoll, kommunikative Fähigkeiten in der medizinischen Ausbildung zu lernen.					
**22**	Meine Fähigkeit, Prüfungen zu bestehen wird mich eher durch das Medizinstudium bringen als meine Fähigkeit zu kommunizieren.					
**23**	Das Erlernen kommunikativer Fähigkeiten lässt sich auf das Erlernen von Medizin anwenden.					
**24**	Es fällt mir schwer, das Erlernen kommunikativer Fähigkeiten ernst zu nehmen.					
**25**	Das Erlernen kommunikativer Fähigkeiten ist wichtig, weil meine Fähigkeit zu kommunizieren eine lebenslange Fertigkeit ist.					
**26**	Das Erlernen kommunikativer Fähigkeiten sollte Psychologiestudenten überlassen werden, nicht Medizinstudenten.					
